# The Role of Medical Societies and the Relevance of Clinical Perspective in the Evolving EU HTA Process: Insights Generated at the 2023 Fall Convention and Survey of the European Access Academy

**DOI:** 10.3390/jmahp12030011

**Published:** 2024-06-22

**Authors:** Elaine Julian, Oriol Solà-Morales, Maria João Garcia, Francine Brinkhuis, Mira Pavlovic, Carlos Martín-Saborido, Robin Doeswijk, Rosa Giuliani, Anne Willemsen, Wim Goettsch, Bernhard Wörmann, Urania Dafni, Heiner C. Bucher, Begoña Pérez-Valderrama, Renato Bernardini, Fabrizio Gianfrate, Carin A. Uyl-de Groot, Jörg Ruof

**Affiliations:** 1 Secretariat of the European Access Academy (EAA), 4059 Basel, Switzerland; 2 HiTT Foundation, International University of Catalonia-UIC, 08015 Barcelona, Spain; 3 F. Hoffmann-La Roche AG, 4070 Basel, Switzerland; 4 Utrecht WHO Collaborating Centre for Pharmaceutical Policy and Regulation, Division of Pharmacoepidemiology and Clinical Pharmacology, Utrecht University, 3584 CS Utrecht, The Netherlands; 5 National Health Care Institute, 1110 AH Diemen, The Netherlands; 6 Medicines Development and Training (MDT) Services, 75020 Paris, France; 7 Ministry of Health, Government of Spain, 28014 Madrid, Spain; 8 European Hematology Association (EHA), 2514 AA The Hague, The Netherlands; 9 Guy’s and St Thomas’ NHS Foundation Trust, London SE1 7EH, UK; 10 German Association of Hematology and Oncology (DGHO), 10178 Berlin, Germany; 11 Division of Hematology, Oncology and Tumor Immunology, Department of Medicine, Charité-Universitätsmedizin Berlin, 13353 Berlin, Germany; 12 European Society for Medical Oncology (ESMO), 6900 Lugano, Switzerland; 13 National and Kapodistrian University of Athens, and Frontier Science Foundation Hellas, 15773 Athens, Greece; 14 Division of Clinical Epidemiology, University Hospital Basel and University of Basel, 4051 Basel, Switzerland; 15 Oncology Department, University Hospital Virgen del Rocío, 41013 Sevilla, Spain; 16 Department of Biomedical and Biotechnological Sciences (BIOMETEC), Section of Pharmacology, University of Catania, 95124 Catania, Italy; 17 University of Ferrara, 44121 Ferrara, Italy; 18 Erasmus School of Health Policy & Management, Institute for Medical Technology Assessment, Erasmus, University Rotterdam, 3062 Rotterdam, The Netherlands; 19 Medical School of Hanover, 30625 Hanover, Germany

**Keywords:** EU HTA, health policy, medical societies, clinical guidelines, best available evidence, health technology assessment

## Abstract

Background: This work aimed to determine the role and action points for the involvement of medical societies in the European Health Technology Assessment (EU HTA) Methods: An online pre-convention survey was developed addressing four areas related to the EU HTA: (i) medical societies’ role; (ii) role of clinical guidelines; (iii) interface with the European Society for Medical Oncology Magnitude of Clinical Benefit Scale (ESMO-MCBS); and (iv) approaching ‘best-available evidence’ (BAE). A descriptive analysis of questionnaire outcomes was conducted to inform the European Access Academy (EAA) Fall Convention 2023. Within the working groups (WGs), action points were identified and prioritised. Results: A total of 57 experts from 15 countries responded to the survey. The WGs were attended by (i) 11, (ii) 10, (iii) 12, and (iv) 12 experts, respectively, representing a variety of national backgrounds and stakeholder profiles. The most relevant action points identified were as follows: (i) incorporation of clinical context into population, intervention, comparator, outcomes (PICO) schemes, (ii) timely provision of up-to-date therapeutic guidelines, (iii) ensuring the inclusion of MCBS insights into the EU HTA process, and (iv) considering randomized controlled trials (RCTs) as the gold standard and leveraging regulatory insights if development programs only include single-arm trials. Conclusions: The involvement of medical societies is a critical success factor for the EU HTA. The identified key action points foster the involvement of patient associations and medical societies.

## 1. Introduction

The European Regulation (EU) 2021/2282 on Health Technology Assessment (EU HTAR) was adopted by the Council and the European Parliament in December 2021 and is effective as of January 2022 [1,2]. The regulation will be applied in a staggered manner starting from January 2025 for joint clinical assessments (JCAs) of both cancer medicines and/or advanced therapy medicinal products (ATMPs), followed by orphan drugs from January 2028 and all other EU-centrally approved medicines from 2030 onward [1,2]. Invasive or implantable high-risk medical devices with “Conformité Européenne” (CE) marking will also be assessed jointly as of January 2025 [1,2].

The EU HTAR aims to harmonise and ensure transparent health technology assessment (HTA) rules and methodological standards, produce high-quality and timely JCAs, and foster adequate and timely collaboration among the European HTA bodies [1,2]. While decisions on the added value (appraisal) and on pricing and reimbursement will remain within the Member States’ remit, the EU HTAR provides a unique opportunity for more efficient use of resources, consolidation of the various national HTA approaches, and ultimately strengthening of the European Health Union [2,3,4,5].

In preparation for the implementation of the EU HTAR (January 2022–January 2025), both the Member States’ Coordination Group on HTA (HTACG) and its respective subgroups, along with a stakeholder network, have now been established [2,6,7]. Furthermore, the European Network for Health Technology Assessment (EUnetHTA) 21—a consortium led by the ‘Zorginstituut’ (ZIN, Diemen, The Netherlands) that included 13 European HTA bodies—has advanced the development of draft methodological and process guidance. This work, conducted under a 24-month service contract agreement awarded by the European Health and Executive Agency (HaDEA), ceased in September 2023 [8,9]. Parallel to the efforts of the European Commission (EC) and the EUnetHTA21 consortium in the preparatory phase, the ‘European Access Academy’ (EAA) was founded in 2021 as a self-organised, crowd-funded, multi-stakeholder initiative, including representatives from academia, patient organisations, medical societies, regulatory and HTA bodies, payers, policy-makers, and health technology developers. The initiative is hosted by Copenhagen University, Utrecht University, Vlerick Business School, Agència de Qualitat I Avaluació Sanitàries de Catalunya, and Erasmus University and endorsed and funded by Springer Healthcare, Abbvie, AstraZeneca, Bayer, Novartis, Pfizer, Roche, Sanofi, vfa, leem, and efpia. The EAA was created with the mission to facilitate and support the development of a joint European value framework for the assessment of innovative health technologies and operates with a steering committee (known as “EAA Faculty”) which was selected as a member of the official EC’s HTA Stakeholder Network in May 2023 [3,4,5,10].

At the inaugural convention of the EAA in May 2022, key process and methodological challenges (uncertainty, patient/intervention/comparators/outcome [PICO], endpoints) were highlighted as warranting further research and resolution to allow for the fulfilment of the intentions of the regulation [5]. Discussions at the EAA’s inaugural convention and at the 2022 Fall Convention further crystallised that successful implementation of the EU HTAR will require the involvement of a wide variety of stakeholders and collaborators, such as patient associations, medical societies, regulators, and health technology developers (HTDs), to achieve high-quality assessments (beyond mere technical discussions) with real value for patients and healthcare providers [4,5,11,12]. Including all relevant stakeholders, particularly medical societies as representatives of the health care providers, is important for the definition of the PICO scheme and in discussions about appropriate methodologies and specific criteria for evaluation within a given clinical context [4,5,13]. The joint European Medicines Agency (EMA)/EUnetHTA work plan (2021–2023) aimed to continue collaboration and facilitate learning between the two institutions, with comprehensive stakeholder involvement being a key topic [4,5,14]. The EUnetHTA21 consortium has also developed guidance documents on the involvement of patients and clinical experts (*D7.2—Guidance on interaction with patients and clinical experts*) and HTD (*D7.1.1–D7.1.3—Interaction between HTA and HTD)*, which might be considered by the HTACG for future adoption [15,16].

**What is the role of PICO in HTA?**

The assessment of the comparative effectiveness and safety of different health technologies, begins with formulating research question(s) that must be answered from a health policy perspective. The PICO framework is a standardised format for translating a policy question into a research question using the following components: population, intervention, comparators and outcomes. Furthermore, the PICO framework will also help to specify the data requirements for the assessment [17].

Evidence-based medicine (EBM) integrates clinical expertise with the best available external clinical evidence to make decisions about the care of individual patients [18]. At the EAA Fall Convention in October 2023, the focus was on the potential role and key contributions of medical societies and clinical experts and the relevance of the clinical perspective in line with EBM practice within the evolving EU HTA framework, with special emphasis on haemato-/oncology medications. In 2023, the EMA adopted 77 positive opinions, with 30 thereof (39%) covering haemato-/oncology conditions, and one ATMP receiving a positive opinion for these types of conditions [19]. Therefore, in addition to being the first field that the EU HTAR will be applied to, haemato-/oncology appears more generally as a pacemaker for innovative research in medicine and for EU HTA, matching the purpose of ‘Europe’s Beating Cancer Plan’ and reflecting the high level of unmet medical need in these fields [20].

Innovative oncology medicines, including ever more targeted interventions for ever-smaller populations, will require innovative methodologies and clinical thinking to determine the additional benefit over the current standard of care [5]. In certain rare conditions as well as for technologies such as ATMPs, it might not be feasible to conduct randomized controlled trials (RCTs) [5]. Therefore, an agreement on alternative options to collect comparative data (‘best-available evidence’) and on the acceptable level of uncertainty in evidence generation in specific disease contexts will be critical to fulfilling the intentions of the EU HTAR as well as ‘Europe’s Beating Cancer Plan’ [5].

The aim of this research was to delve deeply into the potential role of medical societies in EU HTA and identify priority action points to enable systematic and meaningful contributions of medical societies to future EU HTA joint work. 

## 2. Methods

**Figure 1:**
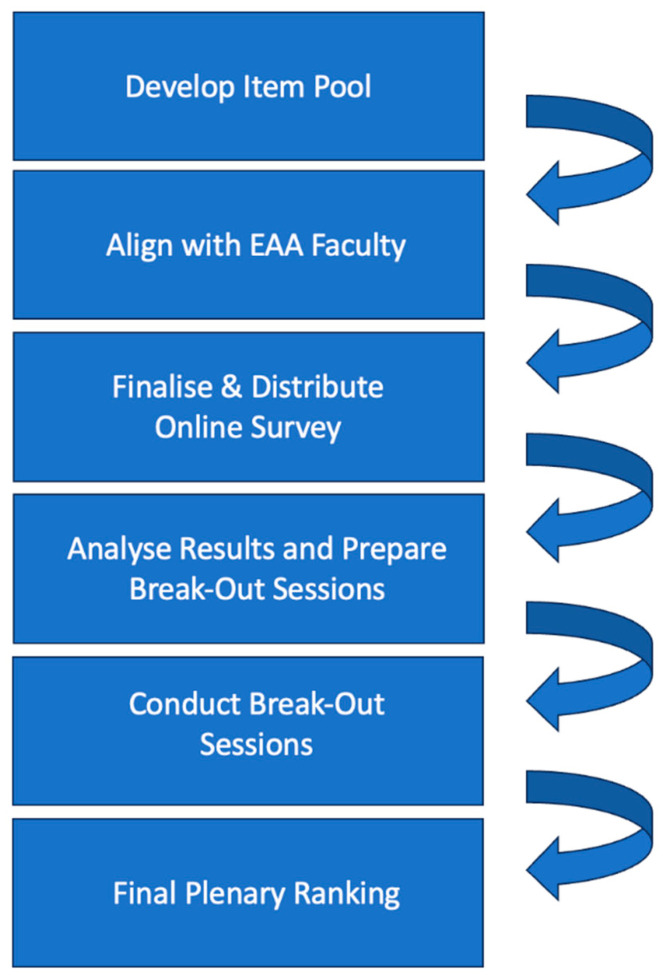
Process flow to determine the role and required action points of medical societies in the European Health Technology Assessment. EAA: European Access Academy.

An overview of the process flow is displayed in Figure 1.

### 2.1. Generation of Input for Discussions through a Pre-Convention Survey

To collect insights on the views of stakeholders regarding the role of medical societies in EU HTA in general as well as on specific aspects of this role, a semi-quantitative research survey was developed, comprising multiple-choice, ranking, and free-text questions (Supplementary Figure S1). For ranking questions, an ordinal Likert response scale (response options: “yes”, “rather yes”, “rather no”, “no”) was utilised for ranking individual items. In addition, free-text questions allowed participants to provide further details and reasoning for their responses. To refine the draft questionnaire, an iterative Delphi process was utilised, conducting two cycles of review by the EAA Faculty in a modified methodology without formal ranking and scoring of the review panel’s responses [21]. The panel consisted of experts from several European countries (Belgium, France, Germany, Italy, Spain, Switzerland, the United Kingdom, and The Netherlands) and institutional backgrounds, including clinicians, patients’ representatives, regulatory authorities, HTA bodies, academia, and HTDs. The survey’s item pool design, validation, and finalization were based on previous EAA practice [3,4,22].

The final online convenience survey was widely distributed between 5 June and 27 October 2023 through multiple channels, including LinkedIn, Twitter, direct e-mail to the EAA network, and platforms, including the EAA website and the EU Health Policy Platform, to receive insights from key stakeholders and collaborators as defined previously [4,10,23].

Responses received through the online survey were pseudonymised and securely stored in a password-protected file before being transferred to an Excel (Version 16) file for analysis. Since the ranking items in the questionnaire were mandatory, no missing data approach was required. For qualitative questions, no imputation was performed as no statistical analyses were conducted on these items. A preliminary analysis of responses was conducted for presentation during the EAA Fall Convention and inclusion in the convention proceedings [24]. After the final data cut (27 October 2023), prespecified descriptive analyses were performed on the quantitative response items. Complete pseudonymised survey data and free-text responses are available upon reasonable request.

### 2.2. Preparation of Break-Out Sessions during the EAA Convention

The 2023 Fall Convention of the EAA was held on 18/19 October 2023 at the Catalan HTA Body, Agència de Qualitat i Avaluació Sanitàries de Catalunya (AQuAS), in Barcelona, Spain. The Convention consisted of plenary sessions as well as break-out sessions with smaller working groups (WGs), both designed as hybrid meetings to allow on-site and remote participation via Zoom. Participation at the EAA Working Session, either on-site or remotely, was open to all representatives of stakeholder groups with relevance in EU HTA (i.e., patients’ and clinicians’ representatives, regulators, HTA body representatives and payers, policy-makers, academics, health technology developers) who registered for the event. In order to achieve a balanced representation, industry participation was limited to a maximum of 15 in total and 2 per company/association. Four dedicated WGs with approximately 15 participants each were constituted in advance, with the following focus topics:

- Medical Societies’ Role in EU HTA (WG 1, Medical Societies);
- Role of clinical guidelines in informing EU HTA scoping and assessment outcomes (WG 2, Clinical Guidelines);
- Interface of the European Society for Medical Oncology–Magnitude of Clinical Benefit Scale (ESMO-MCBS) and HTA (WG 3, Interface with ESMO-MCBS);
- Approaching ‘best-available evidence’ (BAE) for EU HTA (WG 4, Approaching BAE).

The aim of each break-out session was to identify and prioritise a list of activities addressing key aspects of each respective focus topic. Distribution of registered participants among the four WGs was based on the following criteria: (i) personal and professional background, (ii) national diversity in each group, (iii) stakeholder diversity within each group (clinicians’ representatives, patients and patients’ representatives, regulators, HTDs, HTA bodies, payers, policy-makers, and academic representatives), and (iv) participation mode (i.e., on-site vs. remote). It was aimed to have an equal distribution regarding these criteria in all WGs.

In preparation for the break-out sessions, two co-leads and a notetaker were appointed in advance to facilitate each session. Prior to the convention, the EAA secretariat and the leadership teams agreed upon the proposed structure and approach of the break-out sessions to ensure a consistent approach and reporting across all WGs. Co-leads were responsible for facilitating and structuring each of the respective sessions and supported equal involvement of all attendees. Further, the notetaker reported key findings using a predefined PowerPoint template.

**What is ESMO-MCBS and what is it used for?**

It is a standardised, generic, validated approach to stratify the magnitude of clinical benefit that can be anticipated from anti-cancer therapies. It aims to facilitate decision-making, promote accessibility and reduce inequity in accessing high-value cancer treatments [24,25,26,27].

### 2.3. Procedural Approach of the Break-Out Sessions

The parallel break-out sessions were scheduled for 90 min and aimed at facilitating meaningful discussions, encouraging participant input, and generating actionable outcomes to be discussed at the plenary session. After an introductory discussion on the respective topic stimulated and led by the facilitators, each WG developed a comprehensive list of suggested activities pertaining to their respective areas of focus. The identified activities were prioritised by the WG, resulting in a list of four items of the highest priority to be voted on in the subsequent plenary session.

### 2.4. Plenary Session and Ranking

In the final plenary session, the outcomes of the four break-out sessions were reported by the appointed representative of each WG. All attending stakeholders, both on-site and remote, ranked the top four identified action points for each respective WG based on importance. The ranking was facilitated through an online IT-based system [28]. To encourage informed discussions during the session, the aggregated descriptive ranking data were visible to the participants in real time. Key topics were discussed in depth to identify any additional relevant points.

### 2.5. Data Handling and Analysis

Relative ranking data were generated during the plenary session using the online tool Slido [28]. For this, a weight was assigned to each item relative to its position in the ranking list as provided by each respondent, i.e., the highest ranked item had the maximum possible weight, i.e., four, and the lowest ranked item received the lowest weight, i.e., one. Cumulative weights, including all individual responses, were then divided by the total number of respondents for each item, resulting in an average ranked score. To allow for simultaneous ranking by all on-site and remote participants, the ranking questions were shared with the audience via presented QR codes and HTML links.

## 3. Results

### 3.1. Outcomes of the Pre-Convention Survey

**Figure 2:**
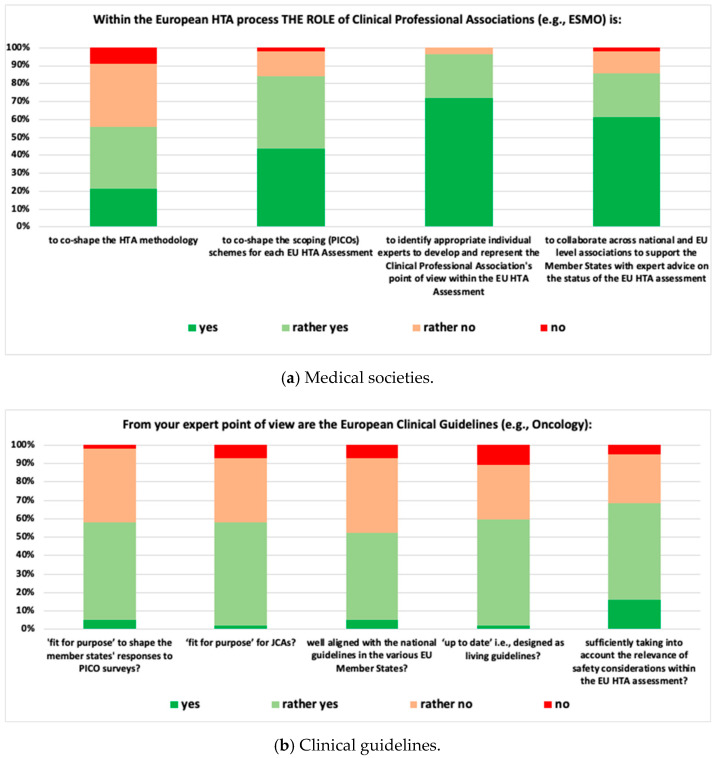

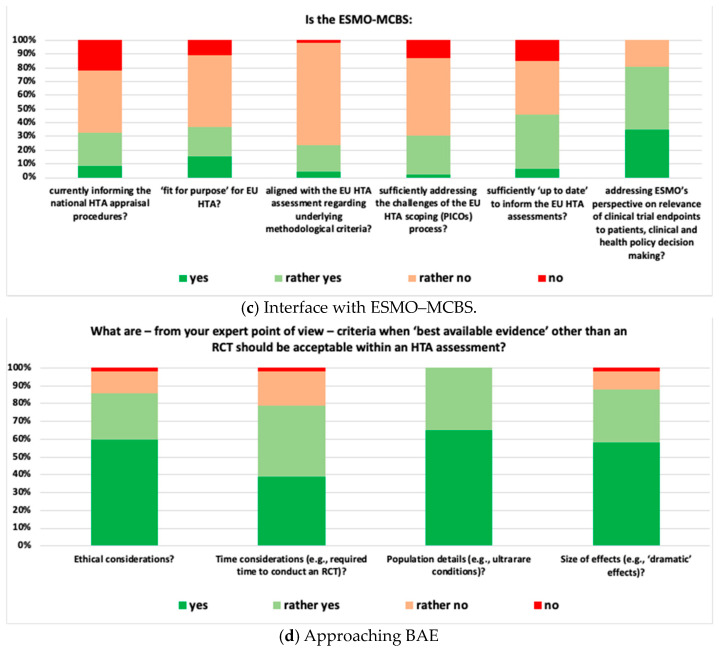
Outcomes of the pre-convention survey. BAE: best-available evidence; ESMO: European Society for Medical Oncology; HTA: health technology assessment; JCA: joint clinical assessment; MCBS: magnitude of clinical benefit scale; PICO: population, intervention, comparator, outcomes; RCT: randomized controlled trial.

A total of 57 experts representing 15 countries and a wide variety of stakeholders (patients, clinicians, regulators, HTDs, HTA bodies, payers, and academia) provided both qualitative and quantitative responses to the pre-convention survey. Responses from key stakeholder groups included: patients’ representatives (n = 2), clinician’s representatives (n = 10), regulators (n = 3), payers (n = 6), and academia (n = 4). Ten respondents did not provide information on which stakeholder group they represented (Supplementary Figure S2a). Moreover, the responses were provided by national, EU-wide, or global representatives and included the following: Global (n = 10), European level (n = 9), Australia (n = 1), Belgium (n = 2); Croatia (n = 1), Egypt (n = 1), France (n = 3), Germany (n = 7), Greece (n = 1), Italy (n = 5), The Netherlands (n = 3); Portugal (n = 2), South Africa (n = 1), Spain (n = 3), Switzerland (n = 4), United Kingdom (n = 3), and United States of America (n = 1) (Supplementary Figure S2b). The outcomes for the four topics are displayed in Figure 2: Medical Societies (Figure 2a), Clinical Guidelines (Figure 2b), Interface with ESMO-MCBS (Figure 2c) and Approaching BAE (Figure 2d). When dichotomizing the findings into yes/rather yes and no/rather no, the highest and lowest-ranked items were as follows:

- Medical Societies: 96.5% of respondents suggested that medical societies should be involved in identifying experts to represent the clinical perspective within the EU HTA Assessment, and 56.1% suggested that medical societies co-shape evidence generation methodology.
- Clinical Guidelines: 68.4% of respondents indicated that clinical guidelines sufficiently take into account the particular clinical context when discussing acceptable safety concerns and 52.6% considered clinical guidelines well aligned with national guidelines.
- Interface with ESMO-MCBS: 80.4% of respondents agreed that the ESMO-MCBS Scorecards adequately address ESMO’s perspective on the relevance of clinical trial endpoints, and 23.9% considered the ESMO-MCBS well aligned with the methodological criteria of the EU HTA assessment.
- Approaching BAE: 78.8% of respondents suggested that, when dealing with high unmet medical need situations, both time considerations and type of evidence should be taken into account to determine when best-available evidence other than an RCT could be acceptable in an HTA assessment. All respondents considered population characteristics (e.g., ultra-rare conditions) as very relevant criteria.

### 3.2. Insights from the Break-Out Sessions

**Figure 3:**
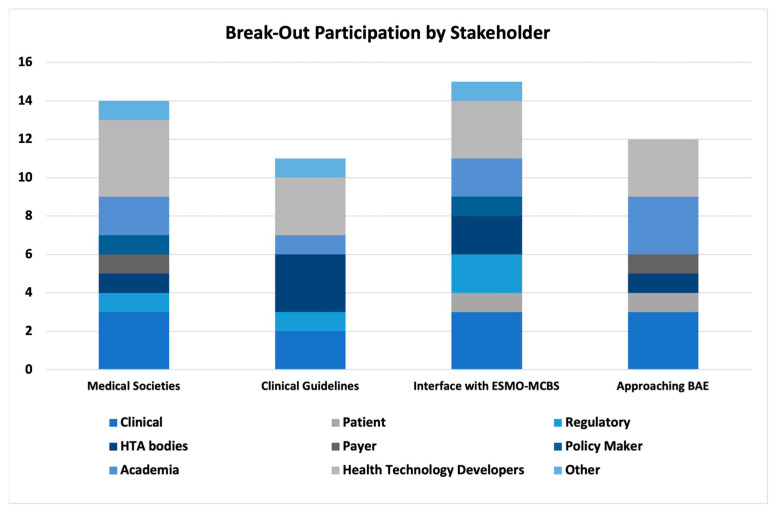
Representation of stakeholder groups in each WG; BAE: best-available evidence; ESMO: European Society for Medical Oncology; HTA: health technology assessment; JCA: joint clinical assessment; MCBS: magnitude of clinical benefit scale; WG: working group.

The break-out sessions were attended by 14 (WG 1, Medical Societies), 11 (WG 2, Clinical Guidelines), 15 (WG 3, Interface with ESMO-MCBS), and 12 (WG 4, Approaching BAE) experts. A variety of national backgrounds (Belgium, France, Germany, Greece, Italy, Malta, The Netherlands, Portugal, Romania, Serbia, Spain, Sweden, Switzerland, and the UK) and stakeholder profiles (Figure 3) were included. Key insights generated for each of the sessions included the following:

- *WG 1:* Within the break-out session, there was a clear consensus that medical societies have an important role to play in EU HTA. However, neither medical societies in general nor the European haematology/oncology societies were perceived as ready to take on that role. Heterogeneity across medical societies, e.g., in terms of organisational structure and resourcing, level of external activities, like involvement in policy shaping and national recommendations, was considered high. Fragmentation into the various subdisciplines, in some cases, lack of an umbrella organisation, limited alignment between national and EU-level societies, lack of established and/or standardised processes to provide timely input into HTA processes, and lack of sufficient and appropriate (i.e., not generating a conflict of interest) funding (the HTA work comes ‘on top of routine clinical work’) were highlighted as major reasons for medical societies not being ready for EU HTA. An overview of the four most relevant contributions of medical societies in the EU HTA process is as agreed in this break-out session provided in Table 1.
- *WG 2:* The general perception within the group was that clinical guidelines are highly relevant for EU HTA but are not yet ‘fit for purpose’ of adequately informing EU HTA. Heterogeneity, e.g., between national treatment guidelines and recommendations on patient management and standard of care for a particular clinical context, lack of timely updates in line with changes in clinical practice, partially limited scope (e.g., not including guidance on biomarker testing) and frequent lack of alignment between European and national level guidelines, particularly when no reference treatment exists, were mentioned as the rationale why clinical guidelines were not perceived ‘fit for purpose’ for EU HTA. Also, break-out participants indicated that so far, guidelines were aimed at ‘bedside’ rather than societal decision-making. To be more useful for EU-level HTA assessment, the process and scope of clinical guidelines may need to be adjusted in order to cover not only adequate PICO choice but also the main aspects of evidence generation for a given clinical condition. Suggested next steps and activities for clinical guidelines are displayed in Table 1.
- *WG 3:* The ESMO-MCBS was perceived as highly relevant but not designed to inform the evolving EU HTA process. Elaboration of the respective MCBS scorecard(s) as well as the underlying scientific rationale occurs once marketing authorisation has been granted—well after the target delivery date of the EU HTA Assessment Report (i.e., ~40 days after granting of marketing authorisation). Moreover, the methodology for the generation of MCBS scorecards and EU HTA methodology differ, and detailed discussion is needed to develop common methodological concepts and define the scope and relevance of the MCBS in the EU HTA process. The scope of EU HTA is broader when compared to the scope of the MCBS; e.g., it reflects by definition a wide variety of treatment standards across the EU. Also, ESMO-MCBS scorecards usually focus on the results of one particular trial rather than reflecting the totality of available evidence. Alignment of a specific MCBS scorecard and the related clinical guidelines was considered key to appropriately informing the EU HTA process. The top priority actions suggested by the WG are displayed in Table 1.
- *WG 4*: The WG agreed (i) that RCTs constitute the ‘gold standard’ for clinical evidence generation, (ii) that evidence other than that derived from RCTs should nevertheless be considered in some specific situations, and (iii) that the concept of ‘totality of evidence’ should be leveraged in the EU HTA assessment. Additional sources of evidence such as single-arm trials, real-world data, post hoc analyses in pre-specified subgroups of patients, indirect treatment comparisons, and systematic reviews were discussed. In particular, when considering a ‘multiplicity’ of European PICO schemes, evidence beyond the primary clinical trial data needs to be taken into account. Contextualization of evidence, considering the unmet medical need, disease characteristics, and size of the eligible patient population, were mentioned as relevant aspects to consider in the EU HTA assessment. Timeline challenges with the EU HTA process were mentioned as a key obstacle in taking an integrative approach to the available evidence. Additionally, clinical data cuts were raised as a challenging topic, i.e., data available for the EU HTA assessment might still be immature, resulting in a potential need for follow-up assessments once more mature data are available. Suggested priority actions are shown in Table 1.

### 3.3. Ranking Obtained in the Final Plenary Session

**Figure 4:**
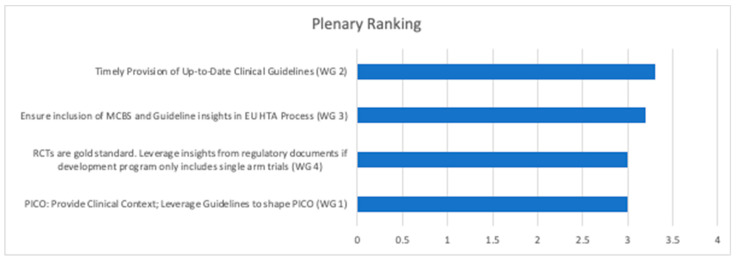
Top priorities as identified by the final plenary ranking as cumulative weighted responses for each list item (range of possible scores: 0–4). HTA: health technology assessment; MCBS: magnitude of clinical benefit scale; PICO: population, intervention, comparator, outcomes; RCT: randomized controlled trial; WG: working group.

The top priority action point from each of the four break-out sessions, as identified in the final plenary ranking, is displayed in Figure 4. Three of those prioritized action points refer to the role of clinical guidelines in the developing EU HTA process, indicating aligned, and up-to-date clinical guidelines as a key priority to inform EU HTA. Further, ensuring clinical input into the PICO schemes and leveraging insights from regulatory documents as a starting point for the assessment of the totality of available evidence if a development program does not include an RCT were considered top priorities during the final panel scoring.

## 4. Discussion

Among the stakeholders and collaborators involved in the EU HTA process an especially important role is the input gathered from clinical experts and medical societies [4,5]. While all involved stakeholders rely on principles of the three pillars of the evidence-based medicine (EBM) triad, including (i) best internal and external evidence, (ii) patient values and expectations, and (iii) clinical experience [29], patients’ and clinicians’ perspectives are needed to balance the technical scope of the assessment of best external evidence. Integrating clinicians’ specific expertise in the clinical context and beyond will be important not only for the JCS and scientific advice but also for the definition of the PICO scheme. Further, their input should be heard in the discussion of appropriate methodologies and specific criteria for evaluation in a given clinical context [4,5]. Here, we evaluated and discussed the potential role of clinicians and medical societies in EU HTA and identified prioritised action points to enable systematic and meaningful contributions of medical societies into future EU HTA joint work. The involvement and input of patient associations has a similarly fundamental role and should be integrated in close coordination with that of medical societies [13]. However, this is not the focus of this work and will be discussed in depth in further work at a later EAA convention.

The importance of involving medical societies in EU HTA was confirmed by the analysis of qualitative pre-convention survey responses as well as the EAA Working Group discussions at the convention, with the main contribution being focused on the clinical context, i.e., the definition of the aim of treatment, unmet medical need, patient-relevance of outcomes, current standard of care in a specific indication. Based on this, a key priority for clinician involvement should be an ‘integrated and pragmatic perspective on PICO design’. However, the principal outcome of discussions was that integrated medical input via coordinated expert networks rather than input from selected individual experts—as stipulated in the regulation—is preferred [2]. Based on the guidance on interaction with patients and clinical experts as developed by EUnetHTA 21, there are a number of challenges to successfully implementing and utilising meaningful clinical input [15]. Among these, appropriate conflict of interest (CoI) management based on the guiding principles of ‘transparency and expertise’ will be crucial to allow for the involvement of representatives with the required expertise and experience [11,30,31]. Further, strong coordination of the relevant medical societies would be needed to facilitate and structure an integrated medical input to address the issue of limited funding.

Clinical guidelines were considered highly relevant for EU HTA joint work; however, they do not substitute direct interaction with clinical experts, and a number of challenges were identified in the survey responses and subsequent WG discussions. Covering the whole diagnostics and treatment pathway of a given condition, they contain integrative information and context as opposed to, e.g., specific HTA questions and decisions. Therefore, guidelines represent the key platform for the communication of structured and integrated medical information, i.e., national treatment standards, in a particular clinical context. Currently, guidelines are designed to guide patient care, not to inform the EU HTA. However, this may need to be revisited to ensure that they are also ‘*fit for purpose*’ in the EU HTA context. In particular, complex structures and processes for guideline updates hinder the timeliness of the medical information presented in the guidelines, a fundamental requirement for HTA [32,33,34,35]. In addition, while in some areas, consolidated European guidelines already exist, in others, a plethora of various national guidelines reflect the differences in health systems and patient access to health technologies [36,37,38,39,40,41,42]. While this heterogeneity poses a challenge for the extraction of relevant information for HTA the development of harmonized guidelines which are useful in different national contexts will be fundamental. Thus, designing such integrative EU guidelines that are updated as soon as new medical information and standards become available may fulfil the purpose of both guiding patient care and informing the HTA, which will further strengthen the EU Health Union [43].

The ESMO-MCBS is a validated and reproducible scale to stratify the magnitude of clinical benefit that can be anticipated from anti-cancer therapies and is applicable across the full range of solid and now also haematological conditions [18,25,26]. It thereby aims to facilitate improved decision-making regarding the value of a new anti-cancer therapy, to promote accessibility and to reduce inequity of access to high-value cancer treatments [27]. These goals align with the aim of the EU HTAR to ‘*improve patient access to live-saving innovative health technologies*’ [1]. Hence, the MCBS was considered by the EAA Working Group a suitable framework for information on the clinical value of oncology treatments across the various stakeholder groups involved in EU HTA. However, it is felt that to achieve optimal utilisation of the MCBS for this purpose, despite the scope of the MCBS Scorecard differing from that of the EU HTA assessment, adjustments might be warranted. Hence, scope and methodology might require further discussion, as well as timing, with the scorecards currently being available only after regulatory approval.

From January 2025, EU HTA procedures will be mandatory for oncology medicines and ATMPs [2]. In these areas, medical advances have led to more targeted research and new treatment paradigms, which are challenging the traditional approaches to clinical research. As a result, RCTs—which are and will remain the unquestionable gold standard for clinical evidence generation—might not be feasible in certain contexts [3,5,44,45,46]. Evidence generated in non-RCTs will inherently carry a level of uncertainty, which needs to be taken into consideration for HTA. While responses to the pre-convention survey and discussions of the EAA WG were partly contradictory in details, there was unanimous agreement that with the changing treatment and research paradigms, it will be crucial to align on how to ‘*manage uncertainty*’ and define ‘*best-available evidence*’, *e.g., taking into account unmet need, incidence and prevalence of the medical condition (i.e., rare or ultra-rare diseases), effect size, timing, ethical questions,* etc., rather than excluding this evidence altogether [3,5]. The key challenge will be the approach to the question of ‘*Who should pay the ‘price’ for increasing levels of uncertainty*’, which will grow in importance with increasing innovation but goes beyond the scope of EU HTA. Consequently, when the first pillar of the EBM Triade (evidence) is weaker due to increased levels of uncertainty, pillars two (patient perspectives) and three (clinical experience) will need to be strengthened to achieve balance [29]. Therefore, strong medical society input will be essential, particularly in those cases, in addition to their important role throughout the EU HTA process.

Additional steps in current procedures might be implemented to further strengthen the integration of clinicians’ as well as patients’ input in national HTA decision-making. The German Association of Haematology and Oncology (DGHO), for example, provides and publishes written statements on clinical context for each assessment of the Federal Joint Committee (G-BA) [47]. A system where such statements by the relevant European medical society, and potentially also one by a respective patient association, are made available to member states together with the JCA report could aid national decision-making. Clinical context and evaluation might thereby provide balance for the information needed for the interpretation of a rather technical JCA report.

### Limitations and Further Research Agenda

This research provides timely and relevant insights into the potential role of medical societies and the relevance of the clinical perspective in future EU HTA processes. Nevertheless, the scope of this research might still be considered exploratory.

Both for the pre-convention survey and for participation in the convention, efforts were made to ensure a broad representation of stakeholder groups and national backgrounds. Invitations to participate were distributed in a multi-channel approach utilising digital/online (e.g., via the EAA website, email, social media, online platforms, and expert networks), as well as conventional methods (e.g., via direct communication, submission of hardcopy survey responses at the convention), and several reminders were published. The number of survey respondents (n = 57) and convention participants (n = 52) was limited, which was, however, not surprising given that in this highly specialized field, most individuals tend to represent the views of their organisations, which are typically formulated and approved through internal processes, rather than expressing their personal perspectives. For the pre-convention survey, all submissions were included in the analysis, so a potential bias due to an imbalance in numbers cannot be ruled out. At the convention, participation was limited to no more than four representatives of one stakeholder per WG, leading to rather balanced WG compositions (Figure 3). Geographical representation covered a variety of European countries and their unique national experiences within each stakeholder group, both in the survey and the convention. Nevertheless, the number of submissions and participants from the Nordics and from Central and Eastern Europe is limited, so their perspectives might not be represented equally with Western Europe in the outcomes and might have introduced some bias in the analysis. Despite this, the geographical coverage and stakeholder representation showcased a broad range of perspectives, indicating the inclusion of diverse viewpoints.

In order to address the limitations as discussed above, future research might, e.g., analyse feedback from a larger sample of respondents, ensure equal representation of stakeholder representatives and national backgrounds, and/or exclude or separately analyse multi-national representatives.

## 5. Conclusions

Evidence-based medicine is founded on the integration of the best available external evidence generated in clinical trials, along with the perspectives of both patients and clinicians. Hence, the involvement of patient associations and medical societies throughout the EU HTA process (JSC as well as JCA) is considered a critical success factor. Key priorities that could be addressed, as identified in this work, are (i) how and when to integrate clinical context in PICOs, (ii) up-to-date ‘agile’ European clinical and methodology guidelines, (iii) adjusted existing tools such as the ESMO-MCBS to be leveraged for EU HTA, and (iv) how to deal with approaching situations in which only non-randomised evidence is included. The establishment of new procedures, including timely input from relevant European patient associations and medical societies to accompany JCA reports, could facilitate the integration of appropriate clinical context in national HTA decision-making.

## Figures and Tables

**Table 1:** Overview of most relevant activities as identified and prioritised in each of the break-out sessions.

Priority	Medical Societies	Clinical Guidelines	Interface with ESMO-MCBS	Approaching BAE
1	PICO: Provide Clinical Context; Leverage Guidelines to shape PICO	Timely provision of Up-to-Date Clinical Guidelines	Ensure inclusion of MCBS and Guideline insights in EU HTA process	RCTs are gold standard. Leverage insights from regulatory documents if development program only includes single arm trials
2	Identification of experts to provide input into EU HTA	Ensure comprehensive Clinical Guidelines (diagnostics; treatment pathways, toxicity)	Include MCBS comparators in EU HTA PICO	Totality of evidence included feed-back from patient organizations should be used
3	PICO: Harmonization of clinical perspective; alignment of EU and national perspective	Integrate and reflect European and national treatment standards in guidelines	Developers to consider leveraging MCBS to inform design of confirmatory trials	Disease Context is critical (e.g., unmet need; ultrarare conditions; poor prognosis etc.)
4	Conflict of Interest (CoI): Contribute to pragmatic and effective CoI management	Adjust purpose of clinical guidelines to serve both HTA and bedside decision making	Consider MCBS tool for national HTA appraisals	Clear guidance re confirmatory follow-up data generation required

CoI: Conflict of interest; ESMO: European Society for Medical Oncology; HTA: health technology assessment; MCBS: magnitude of clinical benefit scale; PICO: population, intervention, comparator, outcomes; RCT: randomized controlled trial.

## Data Availability

Complete pseudonymized survey data and free-text responses are available upon reasonable request.

## Supplementary Materials

The following supporting information can be downloaded at: https://www.mdpi.com/article/10.3390/jmahp12030011/s1, Figure S1: Survey Questions; Figure S2: Pre-convention survey_number of responses per stake-holder and country.
