# Supplementary material for: The Role of Medical Societies and the Relevance of Clinical Perspective in the Evolving EU HTA Process: Insights Generated at the 2023 Fall Convention and Survey of the European Access Academy
Source: J Mark Access Health Policy. 2024 Jun 22;12(3):128–43. doi: 10.3390/jmahp12030011 (PMC11270181; doi:10.3390/jmahp12030011)
Supplement: Supplementary file 1 [file jmahp-12-00011-s001.zip › Supp_Fig_1 Survey Questions.pdf]

Questionnaire - Background Information

1. What country do you represent?

2. Which stakeholder group do you represent? *

- [ ] Patient representative
- [ ] Clinician / Health Care Provider / Medical Association
- [ ] Regulatory
- [ ] HTA body
- [ ] Payer
- [ ] Health Technology Developer (HTD) / HTD Associations
- [ ] Other

Insights on clinical aspects in the context of EU HTA

Please provide input from your point of view:

3. Within the European HTA process **THE ROLE** of Clinical Professional Associations (e.g., ESMO) is: *

	Yes	Rather yes	Rather no	No
to co-shape the HTA methodology	○	○	○	○
to co-shape the scoping (PICOs) schemes for each EU HTA Assessment	○	○	○	○
to identify appropriate individual experts to develop and represent the Clinical Professional Association's point of view within the EU HTA Assessment	○	○	○	○
to collaborate across national and EU level associations to support the Member States with expert advice on the status of the EU HTA assessment	○	○	○	○

4. If there are any further elements of the role of Clinical Professional Associations, please specify

5. From your expert point of view are the **European Clinical Guidelines (e.g., Oncology)**: *

	Yes	Rather yes	Rather no	No
'fit for purpose' to shape the member states' responses to PICO surveys?	○	○	○	○
'fit for purpose' for JCAs?	○	○	○	○
well aligned with the national guidelines in the various EU Member States?	○	○	○	○
'up to date' i.e., designed as living guidelines?	○	○	○	○
sufficiently taking into account the relevance of safety considerations within the EU HTA assessment?	○	○	○	○

6. If there are any further comments on European Clinical Guidelines, please specify

7. Are you aware of the ***ESMO Magnitude of Clinical Benefit Scale (MCBS)***? *

◯ Yes

◯ No

8. Is the **MCBS**: *

	Yes	Rather yes	Rather no	No
currently informing the national HTA appraisal procedures?	○	○	○	○
'fit for purpose' for EU HTA?	○	○	○	○
aligned with the EU HTA assessment regarding underlying methodological criteria?	○	○	○	○
sufficiently addressing the challenges of the EU HTA scoping (PICOs) process?	○	○	○	○
sufficiently 'up to date' to inform the EU HTA assessments?	○	○	○	○
addressing ESMO's perspective on relevance of clinical trial endpoints to patients, clinical and health policy decision making?	○	○	○	○

9. If there are any further comments on the MCBS, please specify

10. What are – from your expert point of view – ***criteria when 'best available evidence' other than an RCT*** should be acceptable within an HTA assessment? *

	Yes	Rather yes	Rather no	No
Ethical considerations?	○	○	○	○
Time considerations (e.g., required time to conduct an RCT)?	○	○	○	○
Population details (e.g., ultrarare conditions)?	○	○	○	○
Size of effects (e.g., 'dramatic' effects)?	○	○	○	○

11. Please provide details for your ratings in the previous question *

12. Can you provide an example where you considered an RCT not applicable within a clinical trial program? Please specify
