# Supplementary material for: The Role of Medical Societies and the Relevance of Clinical Perspective in the Evolving EU HTA Process: Insights Generated at the 2023 Fall Convention and Survey of the European Access Academy
Source: J Mark Access Health Policy. 2024 Jun 22;12(3):128–43. doi: 10.3390/jmahp12030011 (PMC11270181; doi:10.3390/jmahp12030011)
Supplement: Supplementary file 1 [file jmahp-12-00011-s001.zip › Supp_Fig_2 Pre-convention survey_number of responses per stakeholder and country.pdf]

Supp.Fig.2. a-b: Background information on submitted questionnaire responses. (a: Number of respondents per stakeholder group, where provided (10 respondents did not provide information on stakeholder group; b: number of respondents per country or region)

A)

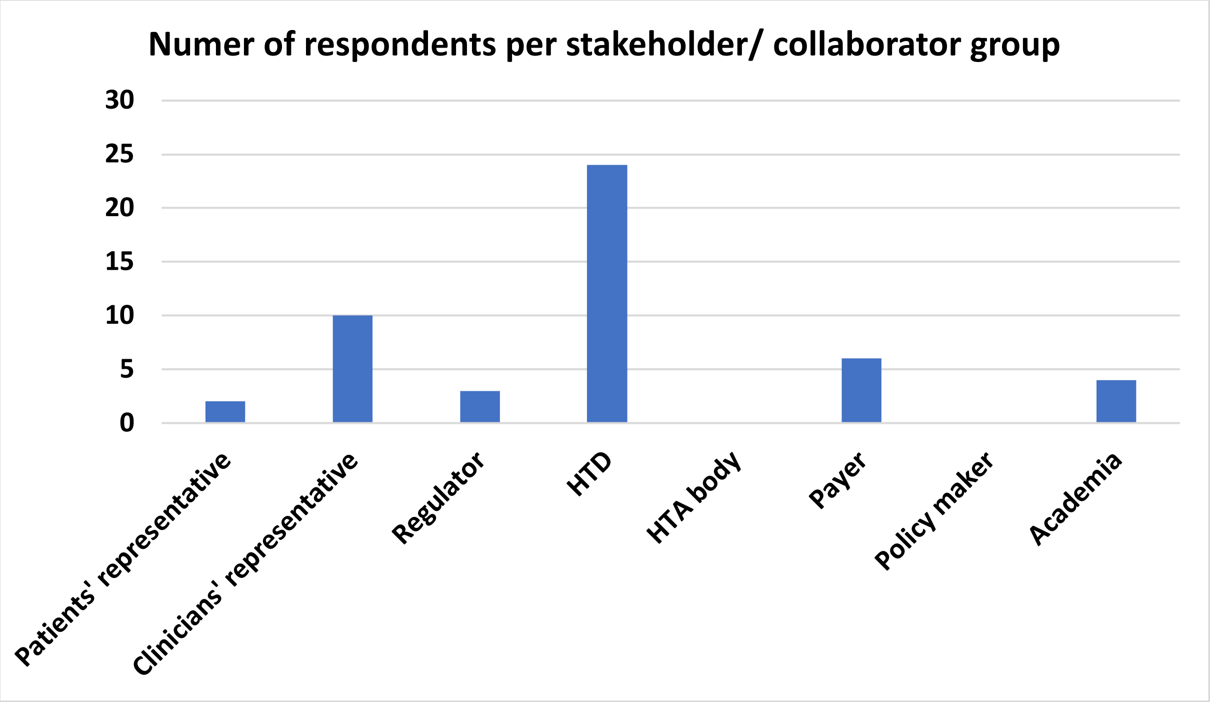

B)

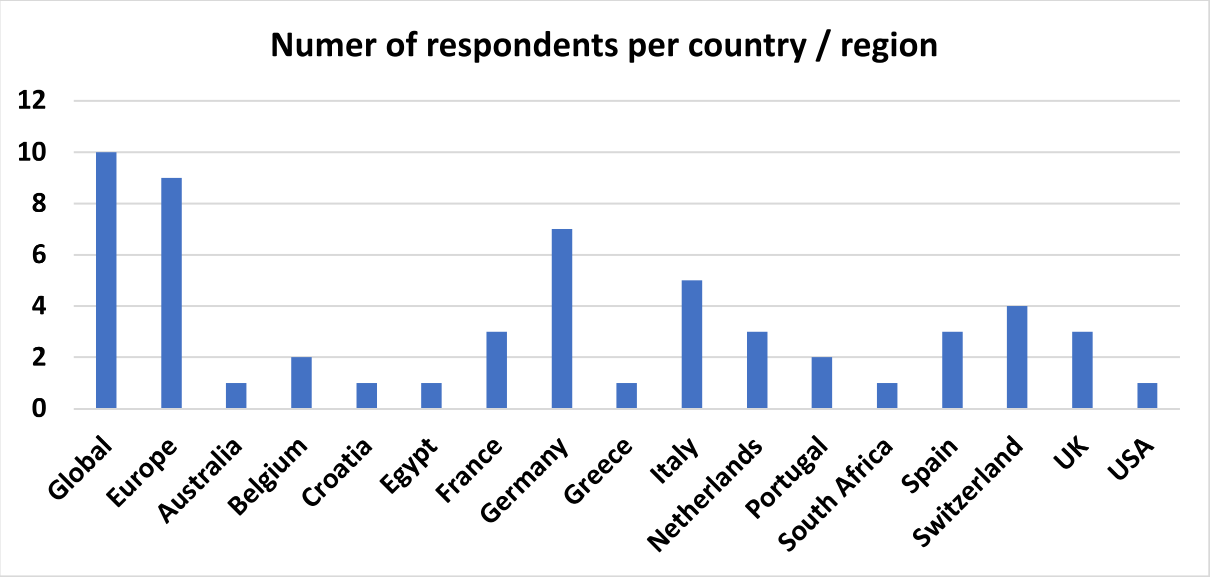

HTA: health technology assessment; HTD: health technology developer; UK: United Kingdom; USA: United States of America
